# Main etiological agents identified in 170 men with urethritis attended at the Fundação Alfredo da Matta, Manaus, Amazonas, Brazil^☆^^☆☆^

**DOI:** 10.1016/j.abd.2020.07.007

**Published:** 2021-01-31

**Authors:** Lucilene Sales de Souza, José Carlos Sardinha, Sinésio Talhari, Marcel Heibel, Mônica Nunes dos Santos, Carolina Talhari

**Affiliations:** a Department of Sexually Transmitted Infections, Fundação Alfredo da Matta, Manaus, AM, Brazil; b Department of Dermatology, Universidade do Estado do Amazonas, Manaus, AM, Brazil; c Department of Dermatology, Fundação Alfredo da Matta, Manaus, AM, Brazil

**Keywords:** *Chlamydia trachomatis*, *Mycoplasma genitalium*, *Mycoplasma hominis*, *Neisseria gonorrhoeae*, Sexually transmitted infections, *Ureaplasma urealyticum*, Urethritis

## Abstract

**Background:**

Sexually transmitted infections (STI) are a global public health problem. Urethritis are among the most common STIs, and can cause several complications and facilitate the transmission of the HIV virus.

**Objectives:**

To investigate the main etiologic agents of urethritis in 170 men treated at Fundação Alfredo da Matta.

**Methods:**

To identify the agents, urethral exudate and urine were collected. Gram and culture tests were performed in Thayer-Martin medium for *Neisseria gonorrhoeae* and polymerase chain reaction for *Neisseria gonorrhoeae, Chlamydia trachomatis, Trichomonas vaginalis, Ureaplasma urealyticum, Ureaplasma parvum, Mycoplasma hominis, Mycoplasma genitalium,* and herpes simplex types 1 and 2.

**Results:**

*N. gonorrhoeae* were identified in 102 (60.0%) patients, *C. trachomatis* in 50 (29.4%), *U. urealyticum* in 29 (17.0%), *M. genitalium* in 11 (6.5 %), *U. parvum* in ten (5.9%), and *M. hominis* in seven (4.1%). Herpes simplex type 2 was diagnosed in 24 (21.6%) of the 111 patients who underwent PCR for this pathogen. In 69 cases there was co-infection; the most frequent were: *N. gonorrhoeae* and *C. trachomatis* in 21 (14.7%) patients; *N. gonorrhoeae* and *C. trachomatis* in 21 (12.4%) patients; *N. gonorrhoeae* and herpes simplex type 2 in 11 (6.5%), and *N. gonorrhoeae* and *U. urealyticum* in nine (5.3%).

**Study limitations:**

Not relevant.

**Conclusion:**

*N. gonorrhoeae, C. trachomatis, U. urealyticum*, and herpes simplex type 2 were the pathogens most frequently identified in the present study. The main coinfection found was *N. gonorrhoeae* and *C. trachomatis*. *T. vaginalis* and herpes simplex type 1 were not identified in any of the patients.

## Introduction

Sexually transmitted infections (STIs) are a global public health problem. According to estimates by the World Health Organization (WHO), more than one million people acquire an STI daily. In 2016, there were 376 million new cases of infection by *Chlamydia trachomatis*, *Neisseria gonorrhoeae*, *Treponema pallidum*, and *Trichomonas vaginalis*.^1^

STIs are contagious diseases, transmitted mainly through sexual intercourse. Among the agents that cause STIs, bacteria, fungi, and protozoa stand out. STIs can be associated with male and female infertility, cancer, pelvic inflammatory disease, prostatitis, epididymitis, and other complications.^1^, ^2^ The importance of STIs as a cofactor in the transmission of the human immunodeficiency virus (HIV) is well known. Several studies have shown the association between urethral discharge, genital ulcers, and condyloma with an increased risk of HIV transmission and changes in the evolution of these infections.^3^, ^4^, ^5^, ^6^, ^7^

Among STIs, urethritis is one of the most common diseases.^1^ In South Africa, it is estimated that, in 2017, there were 1.42 million cases of male urethritis caused by *N. gonorrhoeae* and 1.28 million by *C. trachomatis*.^8^

Urethritis is defined as inflammation of the urethra, whether or not accompanied by urethral exudate and the presence of more than five polymorphonuclear cells per field on the microscopic examination of the smear.^2^ Among the risk factors for urethritis are: age between 20 and 35 years, multiple partners, men who have sex with men (MSM), and individuals with a history of STI.^1^, ^3^, ^4^, ^5^, ^6^, ^7^, ^8^, ^9^

The most common agents of urethritis are *C. trachomatis*, *N. gonorrhoeae, T. vaginalis, Ureaplasma urealyticum,* and *Mycoplasma genitalium*.^2^, ^5^, ^6^, ^9^^,^^10^ *M. hominis, U. parvum*, herpes virus type 2, *Haemophilus influenza*, and adenovirus have also been associated with the etiology of urethritis in men.^11^, ^12^, ^13^

In Brazil, there is little research on the etiology of urethritis. A multicenter study, carried out in Manaus, Recife, Belo Horizonte, São Paulo, and Porto Alegre in 1995 including 473 men, identified (through Gram and culture for gonococcus) *N. gonorrhoeae* in 44% of the samples, *C. trachomatis* in 7%, *N. gonorrhoeae* in association with *C. trachomatis* in 11%, and *T. vaginalis* in 2%.^14^ Ten years later, in a new study with 767 men with STIs residing in São Paulo, Rio de Janeiro, Porto Alegre, Goiânia, Fortaleza, and Manaus, the presence of *N. gonorrhoeae* was identified in 18.4% and *C. trachomatis* in 13.1% of the urine samples assessed through polymerase chain reaction (PCR).^15^

A study conducted in Manaus with 800 male patients with urethral discharge identified *N. gonorrhoeae* in 42.7% of cases, *C. trachomatis* in 10.7%, and infection by both pathogens in 7.3%; in 39.3% of the patients, the etiologic agent was not identified.^16^

In view of the small number of investigations related to urethritis in Brazil, the present study aimed to identify the etiological agents of 170 male patients with urethral discharge. The results of the main aspects related to age, risk factors, etiologic agents, and co-infections are presented

## Methods

This was a descriptive and exploratory cross-sectional study, carried out from November 2015 to May 2016, in patients treated at the Alfredo da Matta Foundation (Fundação Alfredo da Matta [FUAM]), a reference center for STI diagnosis and treatment in the state of Amazonas. Male patients with urethral discharge, without previous antibiotic treatment, were included.

As for the clinical aspects, the guidelines of the Ministry of Health were followed, evaluating the presence of urethral exudate. Data such as age at first sexual intercourse, sexual orientation, number of partners in the last three months, past history of STI, use of medications, and frequency of condom use were also included in the protocol for this project.^2^

After the clinical examination, a digital puncture was performed for the following rapid tests: HIV, syphilis, and hepatitis B and C. In case of positive test for syphilis, blood was collected for venereal disease research laboratory (VDRL).^2^ Rapid HIV tests were performed with the Tri Line kit (Bioclin) and the Immunoblot rapid HIV kit (Biomanguinhos). For rapid testing of syphilis, the Alere syphilis kit (Alere-Abbott) was used; for VDRL, Laborclin reagent was used. The Alere HCV (Alere-Abbott) and Vikia HBsAg (Biomérieux) kits were used for the rapid tests for hepatitis B and C.

On the same day of the patient's first consultation, two urethral exudate samples were collected. This procedure was performed with the Digene kit (hc2 DNA Collection Device). One of the samples was sent to the FUAM laboratory for Gram tests, culture in Thayer-Martin medium, and PCR for herpes simplex (HSV) types 1 and 2. The other sample was sent to the accredited laboratory, in Manaus, to perform PCR for *M. genitalium*, *M. hominis*, *U. urealyticum*, *U. parvum*, *T. vaginalis*, *N. gonorrhoeae*, and *C. trachomatis.*

The urine sample was collected two hours after obtaining the urethral exudate. Patients were instructed to, at the time of urine collection, retract the foreskin, discard the first stream, and collect the midstream. The material was kept refrigerated, at 7 °C, until it was sent to the accredited laboratory.

All patients were treated with azithromycin 1 g, in a single dose, and ciprofloxacin 500 mg, also in a single dose. During the study period, ciprofloxacin was the treatment of choice for *N. gonorrhoeae*.^2^ In cases of persistent symptoms, after seven days, second-line drugs were used, indicated for the treatment of male urethral discharge.^2^

This study was approved by the Research Ethics Committee of FUAM (CAAE 52491215.2.0000.0002). All patients signed an informed consent form.

## Results

The study included 170 patients. The mean age of the patients was 27.7 years, and 96 (57.5%) started sexual life between 15 and 19 years of age. Regarding the number of partners in the last three months, 60 (35.3%) declared that they had only one partner, 56 (32.9%) had two to four, 36 (21.2%), more than five, and 18 (10.6%) reported not having a partner in this period (Table 1).Regarding the frequency of condom use during sexual intercourse, 167 patients answered this questionnaire item: 128 (76.6%) occasionally used them and 32 (19.2%) never used them. Among those who reported sexual orientation, 151 (90.4%) were heterosexual, nine (5.4%) declared themselves MSM, and seven (4.2%) were bisexual (Table 1).

**Table 1:** Epidemiological characteristics of male patients with urethritis seen at the FUAM STI outpatient clinic.

Characteristics	n*	%
Age range		
< 20	31	18.2
20 to 29	80	47.1
30 to 39	38	22.4
40 to 49	17	10.0
> 49	4	2.4
Age at 1st intercourse	(n = 167)	
< 15	66	39.5
15 to 19	96	57.5
20 to 23	5	3.0
		Not reported = 3
Sexual partners in the last 3 months		
None	18	10.6
1	60	35.3
2 to 4	56	32.9
≥ 5	36	21.2
Condom use	(n = 167)	
In all intercourses	7	4.2
Sometimes	128	76.6
Does not use	32	19.2
		Not informed = 3
Sexual orientation	(n = 167)	
Active MSM	1	0.6
Passive MSM	2	1.2
Active/passive MSM	6	3.6
Bisexual	7	4.2
Heterosexual	151	90.4
		Not informed = 3
Venereal history		
Yes	94	55.3
No	76	44.7
		*n = 170

Of the total patients included in the study, 94 (55.3%) reported previous STI, 76 (75.2%) had a history of urethral discharge, 12 (11.9%) reported having had condyloma, eight (7.9 %) reported herpes, three (3.0%) had syphilis, and two (2.0%) had a genital ulcer of unknown etiology.

The Gram test was performed on 152 patients; of these, 94 (61.8%) tested positive for *N. gonorrhoeae*. Culture for *N. gonorrhoeae* was performed in 153 patients; it was positive in 82 (53.6%) cases and negative in 71 (46.4%; Table 2).

**Table 2:** Results of Gram test, culture for *N. gonorrhoeae*, tests for syphilis, HIV, hepatitis B, hepatitis C, and VDRL, in male patients with urethral discharge attended at the FUAM STI outpatient clinic.

Exams	n	Results			
		Positive	%	Negative	%
Gram test	152	94	61.8	58	38.2
Culture for Gonococcus	153	82	53.6	71	46.4
Rapid syphilis test	158	17	10.8	141	89.2
VDRL	23	12	52.2	11	47.8
Rapid HIV test	164	8	4.9	156	95.1
Hepatitis B	142	0	0.0	142	100.0
Hepatitis C	157	1	0.6	156	99.4

The rapid test for syphilis was performed on 158 patients: 17 (10.8%) were reactive. VDRL was performed on 23 patients, 12 (52.2%) of whom presented reactive results.

Among the 164 patients who underwent rapid HIV testing, eight (4.9%) were positive. One (0.6%) of the 157 patients who had serology for hepatitis C was positive, and serology for hepatitis B was negative in all 142 who underwent this test (Table 2).

PCR, performed on urethral exudate, identified *N. gonorrhoeae* in 102 (60.0%) patients, *C. trachomatis* in 50 (29.4%), *U. urealyticum* in 29 (17.0%), *M. genitalium* in 11 (6.5%), *U. parvum* in ten (5.9%), and *M. hominis* in seven (4.1%). *T. vaginalis* was not identified in any of the tested samples. In 23 (13.5%) patients, none of the tested pathogens was identified. PCR for HSV-2 was performed in 111 patients, being positive in 24 (21.6%) samples examined. The frequency of the pathogens identified, alone and in association with others, is listed in Table 3.

**Table 3:** Etiological agents and co-infections identified by PCR in the urethral exudate samples from 170 male patients with urethral discharge attended at the FUAM STI clinic.

Etiological agents	n*	%
*N. gonorrhoeae*	46	27.1
*N. gonorrhoeae* and *C. trachomatis*	21	12.4
*C. trachomatis*	13	7.6
*N. gonorrhoeae* and HSV-2	11	6.5
*N. gonorrhoeae* and *U. urealyticum*	9	5.3
*U. urealyticum*	7	4.1
*M. genitalium*	5	2.9
*U. parvum*	5	2.9
*N. gonorrhoeae, C. trachomatis,* and HSV-2	4	2.4
*C. trachomatis* and *U. urealyticum*	3	1.8
HSV-2	2	1.2
*M. genitalium* and *U. urealyticum*	2	1.2
*N. gonorrhoeae*, *U. parvum,* and HSV-2	2	1.2
*N. gonorrhoeae*, *C. trachomatis,* and *U. urealyticum*	2	1.2
*U. urealyticum* and *M. hominis*	3	1.8
*C. trachomatis* and HSV-2	1	0.6
*C. trachomatis*, *M. hominis,* and *M. urealyticum*	1	0.6
*C. trachomatis*, *M. genitalium*	1	0.6
*C. trachomatis*, *M. genitalium,* and HSV-2	1	0.6
*N. gonorrhoeae*, *M. hominis*, *U. parvum,* and HSV-2	1	0.6
*N. gonorrhoeae* and *M. genitalium*	1	0.6
*N. gonorrhoeae*, *U. parvum,* and *M. hominis*	1	0.6
*N. gonorrhoeae*, *U. urealyticum,* and HSV-2	1	0.6
*N. gonorrhoeae*, *C. trachomatis,* and *M. genitalium*	1	0.6
*N. gonorrhoeae*, *C. trachomatis,* and *M. hominis*	1	0.6
*N. gonorrhoeae*, *C. trachomatis,* and *U. parvum*	1	0.6
*U. urealyticum* and HSV-2	1	0.6
No agent	23	13.5
		*n = 170

In 21 (12.4%) patients, the association of *N. gonorrhoeae* and *C. trachomatis* was identified by PCR in the urethral exudate samples. Thus, 80 (47%) patients had infection by *N. gonorrhoeae* and *C. trachomatis*, either alone or in combination. Eight (4.7%) patients had co-infection of these two microorganisms with a third pathogen (Table 3).

The other most frequent co-infections were: *N. gonorrhoeae* and HSV-2 in 11 (6.5%) patients; *N. gonorrhoeae* and *U. urealyticum* in nine (5.3%); *N. gonorrhoeae*, *C. trachomatis*, and HSV-2 in four (2.4%); and *C. trachomatis* and *U. urealyticum* in three (1.8%; Table 3).

Among the total number of patients included in the study, PCR was performed on the urine samples of 47 patients. *N. gonorrhoeae* was identified in 25 (53.2%) patients, *C. trachomatis* in 11 (23.4%), *U. urealyticum* in three (6.4%), *M. genitalium* in two (4.3%), and *U. parvum* in one (2.1%). The pathogens *M. hominis* and *T. vaginalis* were not identified in any of the samples. In five patients (10.6%), the tests were negative.

In order to verify the agreement rates between conventional microbiological tests, Gram test, and culture for *N. gonorrhoeae* with PCR (in exudate) for the same etiological agent, all 152 and 153 patients who underwent both conventional methods were considered, respectively. Regarding the Gram and PCR test for *N. gonorrhoeae*, a Kappa coefficient of agreement of 0.83 was observed (almost perfect and significant agreement; p < 0.0001). A similar result was found between culture and PCR for *N. gonorrhoeae*: 0.87 (almost perfect and significant agreement; p < 0.0001).

An analysis of the agreement rates between the PCR diagnostic tests in the different clinical samples collected, urethral exudate and urine, was also performed. For this analysis, the 47 patients who underwent both diagnostic methods were considered. Almost perfect and significant agreement was identified for *M. genitalium* (100%) and *N. gonorrhoeae* (87.1%); strong and significant for *C. trachomatis* (68.4%); moderate and significant for *U. urealyticum* (56.1%); and fair for *U. parvum* (56.1%; p < 0.0001).

Among the 170 patients included in the study, 100 (58.8%) were cured with first-line treatment. In three (1.8%) patients, second-line medication was used with clinical resolution; one (0.6%) patient did not respond to first and second line medications, and 66 (38.8%) did not return seven days after administration of the first line treatment, which was considered as treatment abandonment.

No statistical significance was observed in the results of laboratory tests for the three main etiologic agents of urethritis among patients with different sexual orientations.

Regarding the behavior of the four variables referred to as most vulnerable to STIs (age, sexual orientation, number of sexual partners, and venereal history), it was found that age under 30 years was a major risk factor for infection by *N. gonorrhoeae:* odds ratio (OR) = 3.08; 95% confidence interval (CI) (p = 0.001).^2^, ^15^

## Discussion

According to the literature review, this is the first Brazilian study in which PCR was used to identify the main etiologic agents of urethral discharge in men.^13^, ^14^, ^15^

Among the main risk factors for STIs are age between 20 and 35 years, multiple partners, MSM relations, and previous history of STI, especially urethritis^1^, ^2^, ^5^, ^17^ The findings of the present investigation corroborate these observations; most patients reported occasional condom use (76.6%), previous history of STI (55.3%), and more than one sexual partner in the last three months (54.1%).

Coinfection with HIV was diagnosed in eight (4.9%) patients studied. It is known that the presence or past history of urethral discharge increases the chance of HIV transmission in men.^3^, ^4^, ^7^ In Cape Town and Johannesburg, South Africa, the HIV incidence in this population was 23.8% and 38.6%, respectively.^6^ In India, 32.2% of patients were co-infected with HIV at the time of diagnosis of urethral discharge.^9^ In Zimbabwe, 28.5% of men with urethral discharge had HIV; however, among men with genital ulcers, coinfection was higher: 45.2%.^18^ In developed countries, such as Italy, HIV co-infection in men with urethritis was less frequent (4.1%).^19^

In 152 patients, in addition to PCR, a smear was performed to identify Gram-negative diplococci, being positive in 94 (61.8%); 153 were cultured in Thayer-Martin medium, with growth of *N. gonorrhoeae* in 82 (53.4%) cases. It is important to emphasize that, in the present study, without the use of PCR, coinfection with other pathogens would not be diagnosed in 56 (33%) patients, and in 68 (40%) patients the etiologic agent of urethritis would not have been identified.

The etiologic agents of urethral discharge most frequently identified by PCR, alone or in association, were *N. gonorrhoeae* and *C. trachomatis*. These agents were observed in the samples of 131 (77.05%) patients: 46 (27.05%) had only *N. gonorrhoeae* and 13 (7.6%) had only *C. trachomatis*. These results are similar to those reported in India, South Africa, Zimbabwe, and Israel.^5^, ^8^, ^9^, ^20^ In developed countries, such as the United States, Japan, Australia, Estonia, and England, *C. trachomatis* is more frequent than *N. gonorrhoeae.*^9^, ^21^, ^22^, ^23^, ^24^, ^25^, ^26^

The association between *N. gonorrhoeae* and *C. trachomatis* was identified in 12.4% of the studied sample. This finding was higher than those of studies conducted in the United States (5.9%), Israel (5.9%), and Aboriginal communities in Australia (4.1%).^20^, ^21^, ^27^ In turn, this association was found more frequently in Zimbabwe (24.5%) and Italy (30.1%).^5^, ^19^

In the present study, in addition to *N. gonorrhoeae* and *C. trachomatis*, the following pathogens were identified in urethral exudate samples, alone or in association: *U. urealyticum* (29 patients; 17%), *M. genitalium* (11 patients; 6.5%), *U. parvum* (10 patients; 5.9%), and *M. hominis* (seven patients; 4.1%).

Among the 170 patients in the present study, other co-infections were identified, such as *N. gonorrhoeae* and HSV-2 (11 patients; 6.5%), *N. gonorrhoeae* and *U. urealyticum* (nine patients; 5.3%), *N. gonorrhoeae, C. trachomatis* and HSV-2 (four patients; 2.4%), and *C. trachomatis* associated with *U. urealyticum* (three patients; 1.8%). Associations of *T. vaginalis* with *N. gonorrhoeae* and C. *trachomatis*; *M. genitalium* and *N. gonorrhoeae*; *M. genitalium* and C. *trachomatis;* and *C. trachomatis* and *U. urealyticum* have already been demonstrated in cases of urethral discharge.^21^, ^27^, ^28^, ^29^

Bacteria of the *Mycoplasma* and *Ureaplasma* genera have been identified as important causes of non-gonococcal urethritis (NGU), particularly *M. genitalium* and *U. urealyticum*.^8^, ^28^, ^30^, ^31^, ^32^, ^33^, ^34^ Regarding the other species of *Mycoplasma* and *Ureaplasma*, doubts about their etiopathogenic role in urethritis still persist.^33^, ^35^

In studies with groups of urethritis and healthy patients, *U. parvum* and *M. hominis* were identified in both groups, in similar proportions.^28^, ^32^ Despite the ambiguity of the etiopathogenic role of *U. parvum*, a study carried out with symptomatic and asymptomatic patients, with positive PCR for this pathogen in the urine found a positive correlation between the number of *U. parvum* 16S rRNA gene copies and polymorphonuclear count in the urine.^33^ In the present study, it was not possible to determine whether *M. hominis* and *U. parvum* were actually causes of urethritis. It is important to note that *U. parvum* and *M. hominis* are part of the urethra microbiota.^30^, ^33^ Therefore, further studies are needed.

Among the 111 patients with urethral discharge who underwent PCR for HSV-2, this test was positive in 24 (21.6%) patients, alone or in association with other pathogens. In the literature, there are reports that these patients tend to have inflammation of the urinary meatus, dysuria, genital ulceration, and inguinal lymphadenopathy; the presence of urethral exudate is less common when compared to patients with urethritis caused by *C. trachomatis*.^36^, ^37^

The non-identification of *T. vaginalis* by PCR, among the samples of the 170 investigated patients, is noteworthy. In Malawi, this pathogen was identified in 17.3% of the cases studied.^38^ In Japan, England, and Brazil, *T. vaginalis* is rarely identified in patients with urethritis.^9^, ^14^, ^39^ A result similar to the present study was reported in Israel, by Gottemann et al.; in that study, no cases of urethral discharge caused by *T. vaginalis* were identified.^20^ The etiopathogenic role of this pathogen in urethritis is controversial.

Currently, ceftriaxone and azithromycin are used in Brazil as a first-line treatment for urethral discharge in male patients. The first drug has an effect on *N. gonorrhoeae* and the second on *C. trachomatis*, pathogens identified in 131 (77%) patients in the present study.^2^ It should be noted that azithromycin also acts on *U. urealyticum* and *U. parvum*.^40^ In the present study, these two bacteria were identified in 13 (7.6%) patients. Therefore, the combination of drugs used in the study would, theoretically, be effective in more than 84% of the present sample. In view of the high rate of patients who did not return after treatment (38.8%), this finding could not be confirmed.

The choice of the urethritis diagnostic method depends on the context in which the patient is treated. However, when available, the PCR exam would be ideal because, in the same material, other agents that may be associated with *N. gonorrhoeae* or which may cause NGU by themselves can be investigated. Culture in Thayer-Martin medium is also important for the identification of *N. gonorrhoeae*, particularly in cases of diagnostic doubt.

Among the patients who underwent PCR on urethral exudate and urine samples, the agreement between the results was almost perfect and significant in the identification of the pathogens *N. gonorrhoeae* and *M. genitalium*, and strong for *C. trachomatis*. Urine PCR has been performed in several centers and is a feasible test when there is no evident urethral exudate.^30^, ^33^, ^35^

## Conclusions

The findings of the present study demonstrate that molecular biology exams, such as PCR, are important for the rapid and accurate diagnosis of the etiologic agents of gonococcal and non-gonococcal urethritis. These procedures also allow the diagnosis of co-infections and adequate treatment of patients who do not respond to the recommendations in the flowcharts for the syndromic approach to urethral discharge.

In the present study, the presence of *N. gonorrhoeae* and *C. trachomatis*, isolated or in association, was observed in 47% of the samples. In 77% of the samples, these agents were identified separately, in association with each other and with other pathogens, which justifies the maintenance of ceftriaxone and azithromycin as first-line drugs in the therapeutic scheme of urethral discharge in Brazil. In view of the increase in cases of *N. gonorrhoeae* resistance to ciprofloxacin, this antibiotic was replaced by ceftriaxone in 2018.^2^

The findings of this study indicate that there is no need for treatment for *T. vaginalis* in cases of therapeutic failure with the syndromic approach, as it was not isolated in any of the patients. Further studies are needed to confirm this finding.

## Ethical aspects

This study was approved by the Research Ethics Committee of FUAM (CAAE 52491215.2.0000.0002). All patients signed an informed consent form. All patients included signed an informed consent.

## Financial support

None declared.

## Authors’ contributions

Lucilene Sales de Souza: Conception and study design; survey of data; analysis and interpretation of data; drafting of the article; critical review of intellectual content; final approval of the version to published.

José Carlos Sardinha: Conception and design of the study; analysis and interpretation of data; critical review of intellectual content; final approval of the version to be published.

Sinésio Talhari: Conception and design of the study; analysis and interpretation of data; drafting of the article; critical review of intellectual content; final approval of the version to be published.

Marcel Heibel: Survey of data; analysis and interpretation of data; critical review of intellectual content; final approval of the version to be published.

Mônica Nunes dos Santos: Analysis and interpretation of data; critical review of intellectual content; final approval of the version to be published.

Carolina Talhari: Conception and design of the study; analysis and interpretation of data; drafting of the article; critical review of intellectual content; final approval of the version to be published.

## Conflicts of interest

The Sabin laboratory performed the PCR exams for the detection of *M. genitalium, Mycoplasma hominis, U. urealyticum, Ureaplasma parvum, T. vaginalis, N. gonorrhoeae,* and *C. trachomatis*.
